# Synchrotron study of poly[[di-μ-aqua­(μ-2,2′-bipyridyl-5,5′-dicarboxyl­ato)di­potassium] dihydrate]

**DOI:** 10.1107/S1600536809038756

**Published:** 2009-09-30

**Authors:** Jeffrey A. Bertke, Allen G. Oliver, Kenneth W. Henderson

**Affiliations:** a University of Notre Dame, Department of Chemistry and Biochemistry, 251 Nieuwland Science Hall, Notre Dame, IN 46556-5670, USA

## Abstract

The title compound, {[K_2_(C_12_H_6_N_2_O_4_)(H_2_O)_2_]·2H_2_O}_*n*_, forms a three-dimensional coordination polymer in the solid state. The asymmetric unit consists of one K^+^ ion, half of a 2,2′-bipyridyl-5,5′-dicarboxyl­ate ligand, one coordinated water mol­ecule and one solvent water mol­ecule. The K^+^ ion is 7-coordinated by the oxygen atoms of two water mol­ecules and by five oxygen atoms of four carboxyl­ate groups, one of which is chelating. The extended structure can be described as a binodal network in which each K^+^ is a six-connected node, bonding to four carboxyl­ate groups and two bridging water mol­ecules, and the 2,2′-bipyridyl-5,5′-dicarboxyl­ate linkers are eight-connected nodes, with each carboxyl­ate group bridging four metal centers. Overall, this arrangement generates a complex network with point symbol {3^4^.4^12^.5^12^}{3^4^.4^4^.5^4^.6^3^}_2_. Both of the bridging water mol­ecules participate as donors in hydrogen-bonding inter­actions; one to solvent water mol­ecules and a second to an oxygen atom of a carboxyl­ate group.

## Related literature

For topological analysis, see: Blatov (2007 ▶). For background to metal-organic frameworks (MOFs), see: MacDougall *et al.* (2005 ▶). The 2,2′-bipyridyl-5,5′-dicarboxyl­ate ligand has been used as a linear linker for a variety of MOFs, see: Finn & Zubieta, (2002 ▶); Schoknecht & Kempe (2004 ▶); Szeto *et al.*, (2008 ▶). It is a particularly inter­esting linker due to the fact that the pyridyl N atoms have the ability to act as Lewis bases for binding metal centers (Szeto *et al.*, 2008 ▶). There has been only one structural example of this ligand bound to an alkali metal reported, *viz.* Rb (Hafizovic *et al.*, 2007 ▶). For synthetic details, see: Anderson *et al.* (1985 ▶).
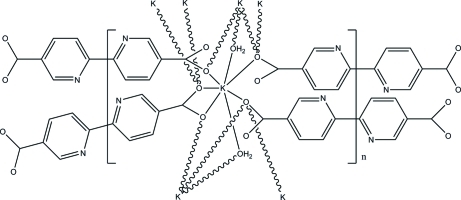


## Experimental

### 

#### Crystal data

[K_2_(C_12_H_6_N_2_O_4_)(H_2_O)_2_]·2H_2_O
*M* *_r_* = 392.46
Monoclinic, 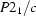

*a* = 3.6769 (6) Å
*b* = 8.2042 (14) Å
*c* = 26.292 (4) Å
β = 92.924 (2)°
*V* = 792.1 (2) Å^3^
*Z* = 2
Synchrotron radiation
λ = 0.77490 Å
μ = 0.76 mm^−1^
*T* = 150 K
0.04 × 0.03 × 0.01 mm

#### Data collection

Bruker APEXII diffractometer
Absorption correction: multi-scan (*SADABS*; Sheldrick, 2008*a* ▶) *T*_min_ = 0.654, *T*_max_ = 0.746
8561 measured reflections
1622 independent reflections
1301 reflections with *I* > 2σ(*I*)
*R*_int_ = 0.045

#### Refinement

*R*[*F* ^2^ > 2σ(*F* ^2^)] = 0.045
*wR*(*F* ^2^) = 0.128
*S* = 1.08
1622 reflections
117 parameters
H atoms treated by a mixture of independent and constrained refinement
Δρ_max_ = 1.03 e Å^−3^
Δρ_min_ = −0.51 e Å^−3^

### 

Data collection: *APEX2* (Bruker, 2008 ▶); cell refinement: *SAINT* (Bruker, 2008 ▶); data reduction: *SAINT*; program(s) used to solve structure: *SHELXS97* (Sheldrick, 2008*b* ▶); program(s) used to refine structure: *SHELXL97* (Sheldrick, 2008*b* ▶); molecular graphics: *SHELXTL* (Sheldrick, 2008*b* ▶); software used to prepare material for publication: *SHELXTL*.

## Supplementary Material

Crystal structure: contains datablocks global, I. DOI: 10.1107/S1600536809038756/wm2256sup1.cif

Structure factors: contains datablocks I. DOI: 10.1107/S1600536809038756/wm2256Isup2.hkl

Additional supplementary materials:  crystallographic information; 3D view; checkCIF report

## Figures and Tables

**Table 1:** Hydrogen-bond geometry (Å, °)

*D*—H⋯*A*	*D*—H	H⋯*A*	*D*⋯*A*	*D*—H⋯*A*
O3—H3*A*⋯O1^i^	0.84 (5)	1.93 (5)	2.770 (4)	177 (5)
O3—H3*B*⋯O4^ii^	0.84 (6)	2.15 (6)	2.976 (5)	169 (5)

Symmetry codes: (i) 
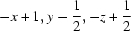
; (ii) 
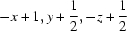
.

**Fig. 1.**
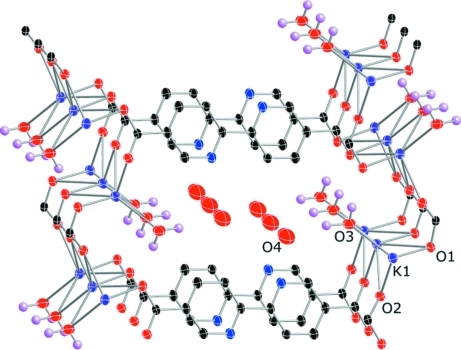
A portion of the polymeric structure of the title compound. All hydrogen atoms except those located on O3 have been omitted. Thermal ellipsoids are shown at the 50% probablility level.

**Fig. 2.**
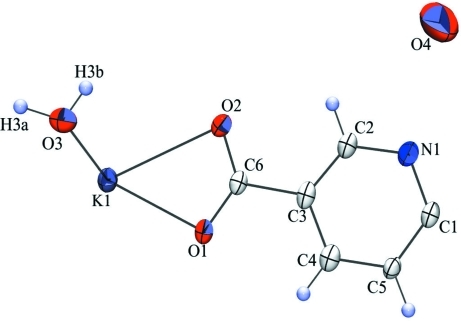
Thermal ellipsoid plot of the asymmetric unit of the title compound. Thermal ellipsoids are shown at the 50% probablility level.

## supplementary crystallographic information

### Comment

We are interested in using pre-assembled and structurally well defined aggregates as secondary building units (SBUs) to build coordination polymers or metal-organic frameworks (MOFs) (MacDougall *et al.*, 2005). The title compound was synthesized as a precursor for use as a linker in the construction of MOFs. The 2,2'-bipyridyl-5,5'-dicarboxylate ligand has been used as a linear linker for a variety of MOFs (Finn & Zubieta, 2002; Schoknecht & Kempe, 2004); Szeto *et al.*, 2008). It is a particularly interesting linker due to the fact that the pyridyl N atoms have the ability to act as Lewis bases for binding metal centers (Szeto *et al.*, 2008).

The asymmetric unit consists of one K^+^ ion, half of a 2,2'-bipyridyl-5,5'-dicarboxylate ligand, one coordinated water molecule and one solvent water molecule (Fig. 1). The extended crystal structure of the title compound is composed of two-dimensional sheets consisting of potassium-carboxylate and potassium-water interactions. These sheets extend parallel to (001). The three-dimensional assembly is completed by connection of the sheets through the 2,2'-bipyridyl linkers (Fig. 2). Small channels run along the *a* axis and contain the disordered solvent water molecules. The geometry around the seven-coordinate potassium metal center can be described as a distorted monocapped trigonal-prism. The faces deviate from the ideal triangular, with angles of 49.69°(O1—O3—O2), 61.19° (O2—O1—O3) and 69.10° (O1—O2—O3). There is also a short contact distance of 2.826 (6) Å between O4 and N1, which is most likely due to a hydrogen bonding interaction. However, the hydrogen atoms on the solvent water molecule could not be located in the difference map.

For the topological analysis of the structure, the program *TOPOS* was used (Blatov, 2007). There has been only one structural example of the 2,2'-bipyridyl-5,5'-dicarboxylate ligand bound to an alkali metal reported, *viz.* Rb (Hafizovic *et al.*, 2007).

### Experimental

The title complex was prepared using a modification of the method of Seddon and Pilling (Anderson *et al.*, 1985) for the preparation of 2,2'-bipyridyl-4,4'-dicarboxylic acid. 5,5'-dimethyl-2,2'-bipyridine and K[MnO_4_] were purchased from Aldrich and used without further purification. ^1^H NMR data were collected on a Varian Unity Plus 300 spectrometer at 298 K. K[MnO_4_] (10 g, 63 mmol) was added to a solution of 5,5'-dimethyl-2,2'-bipyridine (1.6 g, 8.7 mmol) in 100 mL H_2_O. The mixture was heated to reflux for 12 h. Upon cooling, the mixture was filtered and the black precipitate was washed with water (2*x* 30 mL). The filtrate and the washings were combined and extracted with diethyl ether to remove unreacted starting material. The aqueous fraction was collected and the solvent was removed under vacuum, leaving a sticky white solid. The solid was heated under vacuum at 373 K overnight to give a fine white powder. Single crystals were grown from an aqueous solution upon slow evaporation of the solvent.

### Refinement

Hydrogen atoms were placed at calculated positions and allowed to ride on the position of the parent atom, with the exception of those on the coordinated O3 water molecule which were located in the difference fourier map. H atoms of the uncoordinated water molecule O4 could not be located unambiguously and were eventually excluded from the refinement.

### Crystal data

**Table:**

[K_2_(C_12_H_6_N_2_O_4_)(H_2_O)_2_]·2H_2_O	*F*(000) = 404
*M**_r_* = 392.46	*D*_x_ = 1.646 Mg m^−^^3^
Monoclinic, *P*2_1_/*c*	Synchrotron radiation, λ = 0.77490 Å
Hall symbol: -P 2yc	Cell parameters from 1984 reflections
*a* = 3.6769 (6) Å	θ = 2.8–27.6°
*b* = 8.2042 (14) Å	µ = 0.76 mm^−^^1^
*c* = 26.292 (4) Å	*T* = 150 K
β = 92.924 (2)°	Plate, colorless
*V* = 792.1 (2) Å^3^	0.04 × 0.03 × 0.01 mm
*Z* = 2	

### Data collection

**Table:**

Bruker APEXII diffractometer	1622 independent reflections
Radiation source: synchrotron	1301 reflections with *I* > 2σ(*I*)
channel-cut Si-<111> crystal	*R*_int_ = 0.045
Detector resolution: 83.33 pixels mm^-1^	θ_max_ = 29.0°, θ_min_ = 2.8°
ω and φ scans	*h* = −4→4
Absorption correction: multi-scan (*SADABS*; Sheldrick, 2008*a*)	*k* = −10→10
*T*_min_ = 0.654, *T*_max_ = 0.746	*l* = −32→32
8561 measured reflections	

### Refinement

**Table:**

Refinement on *F*^2^	Primary atom site location: structure-invariant direct methods
Least-squares matrix: full	Secondary atom site location: difference Fourier map
*R*[*F*^2^ > 2σ(*F*^2^)] = 0.045	Hydrogen site location: inferred from neighbouring sites
*wR*(*F*^2^) = 0.128	H atoms treated by a mixture of independent and constrained refinement
*S* = 1.08	*w* = 1/[σ^2^(*F*_o_^2^) + (0.0609*P*)^2^ + 1.2501*P*] where *P* = (*F*_o_^2^ + 2*F*_c_^2^)/3
1622 reflections	(Δ/σ)_max_ < 0.001
117 parameters	Δρ_max_ = 1.03 e Å^−^^3^
0 restraints	Δρ_min_ = −0.51 e Å^−^^3^

### Special details

**Table:**

Geometry. All e.s.d.'s (except the e.s.d. in the dihedral angle between two l.s. planes) are estimated using the full covariance matrix. The cell e.s.d.'s are taken into account individually in the estimation of e.s.d.'s in distances, angles and torsion angles; correlations between e.s.d.'s in cell parameters are only used when they are defined by crystal symmetry. An approximate (isotropic) treatment of cell e.s.d.'s is used for estimating e.s.d.'s involving l.s. planes.
Refinement. Refinement of *F*^2^ against ALL reflections. The weighted *R*-factor *wR* and goodness of fit *S* are based on *F*^2^, conventional *R*-factors *R* are based on *F*, with *F* set to zero for negative *F*^2^. The threshold expression of *F*^2^ > σ(*F*^2^) is used only for calculating *R*-factors(gt) *etc*. and is not relevant to the choice of reflections for refinement. *R*-factors based on *F*^2^ are statistically about twice as large as those based on *F*, and *R*- factors based on ALL data will be even larger.

### Fractional atomic coordinates and isotropic or equivalent isotropic displacement parameters (Å^2^)

**Table:**

	*x*	*y*	*z*	*U*_iso_*/*U*_eq_	
K1	0.06233 (18)	0.72796 (8)	0.22289 (2)	0.0250 (2)	
O1	0.5909 (6)	0.7368 (3)	0.30066 (8)	0.0257 (5)	
O2	0.4216 (6)	0.4762 (3)	0.29578 (8)	0.0262 (5)	
O3	0.5493 (7)	0.5466 (3)	0.16339 (11)	0.0359 (6)	
H3A	0.499 (13)	0.454 (6)	0.1748 (18)	0.052 (14)*	
H3B	0.528 (16)	0.539 (7)	0.132 (2)	0.080 (19)*	
O4	0.537 (2)	0.0661 (6)	0.44962 (17)	0.154 (3)	
N1	0.7659 (7)	0.3947 (3)	0.44667 (10)	0.0272 (6)	
C1	0.9350 (8)	0.5168 (4)	0.47310 (10)	0.0216 (6)	
C2	0.6527 (8)	0.4234 (4)	0.39788 (11)	0.0251 (7)	
H2A	0.5352	0.3375	0.3792	0.030*	
C3	0.6981 (8)	0.5704 (4)	0.37364 (11)	0.0225 (6)	
C4	0.8744 (8)	0.6938 (4)	0.40162 (11)	0.0252 (7)	
H4A	0.9138	0.7968	0.3863	0.030*	
C5	0.9916 (8)	0.6669 (4)	0.45143 (11)	0.0209 (6)	
H5A	1.1106	0.7510	0.4707	0.025*	
C6	0.5582 (8)	0.5960 (4)	0.31898 (11)	0.0200 (6)	

### Atomic displacement parameters (Å^2^)

**Table:**

	*U*^11^	*U*^22^	*U*^33^	*U*^12^	*U*^13^	*U*^23^
K1	0.0231 (3)	0.0293 (4)	0.0222 (4)	−0.0001 (3)	−0.0021 (2)	0.0022 (3)
O1	0.0312 (12)	0.0272 (12)	0.0182 (10)	0.0001 (9)	−0.0035 (9)	0.0015 (8)
O2	0.0310 (12)	0.0258 (12)	0.0209 (10)	−0.0008 (9)	−0.0053 (9)	−0.0028 (9)
O3	0.0432 (15)	0.0268 (14)	0.0371 (16)	−0.0043 (11)	−0.0035 (12)	0.0001 (11)
O4	0.345 (9)	0.055 (3)	0.059 (3)	0.017 (4)	−0.009 (4)	−0.001 (2)
N1	0.0293 (14)	0.0311 (15)	0.0209 (13)	−0.0025 (11)	−0.0025 (11)	−0.0005 (11)
C1	0.0182 (13)	0.0305 (16)	0.0159 (14)	0.0023 (12)	−0.0013 (11)	−0.0020 (12)
C2	0.0250 (16)	0.0316 (17)	0.0183 (14)	0.0008 (13)	−0.0024 (12)	−0.0005 (12)
C3	0.0176 (14)	0.0330 (16)	0.0168 (14)	0.0038 (12)	0.0003 (11)	−0.0008 (12)
C4	0.0225 (15)	0.0325 (17)	0.0205 (15)	0.0018 (12)	−0.0003 (11)	−0.0004 (12)
C5	0.0200 (14)	0.0256 (15)	0.0167 (14)	−0.0018 (11)	−0.0040 (11)	−0.0009 (11)
C6	0.0181 (14)	0.0261 (16)	0.0158 (14)	0.0029 (11)	0.0002 (11)	−0.0014 (11)

### Geometric parameters (Å, °)

**Table:**

K1—O2^i^	2.732 (2)	N1—C2	1.349 (4)
K1—O1^ii^	2.748 (2)	N1—C1	1.353 (4)
K1—O1	2.749 (2)	C1—C5	1.376 (4)
K1—O3^ii^	2.814 (3)	C1—C1^iv^	1.496 (6)
K1—O2^iii^	2.843 (2)	C2—C3	1.378 (5)
K1—O3	2.855 (3)	C2—H2A	0.9500
K1—O2	3.070 (2)	C3—C4	1.392 (4)
O1—C6	1.259 (4)	C3—C6	1.516 (4)
O2—C6	1.249 (4)	C4—C5	1.376 (4)
O3—H3A	0.84 (5)	C4—H4A	0.9500
O3—H3B	0.84 (6)	C5—H5A	0.9500
			
O2^i^—K1—O1^ii^	71.60 (7)	K1^vi^—O2—K1	97.69 (6)
O2^i^—K1—O1	122.90 (7)	K1^vii^—O2—K1	130.08 (8)
O1^ii^—K1—O1	83.96 (6)	K1^v^—O3—K1	80.87 (7)
O2^i^—K1—O3^ii^	83.24 (8)	K1^v^—O3—H3A	116 (3)
O1^ii^—K1—O3^ii^	89.84 (8)	K1—O3—H3A	97 (3)
O1—K1—O3^ii^	148.85 (8)	K1^v^—O3—H3B	129 (4)
O2^i^—K1—O2^iii^	82.49 (6)	K1—O3—H3B	124 (4)
O1^ii^—K1—O2^iii^	124.15 (7)	H3A—O3—H3B	106 (5)
O1—K1—O2^iii^	69.93 (6)	C2—N1—C1	118.1 (3)
O3^ii^—K1—O2^iii^	135.82 (8)	N1—C1—C5	121.7 (3)
O2^i^—K1—O3	135.30 (8)	N1—C1—C1^iv^	117.7 (3)
O1^ii^—K1—O3	149.13 (8)	C5—C1—C1^iv^	120.6 (3)
O1—K1—O3	88.96 (7)	N1—C2—C3	123.6 (3)
O3^ii^—K1—O3	80.87 (7)	N1—C2—H2A	118.2
O2^iii^—K1—O3	80.56 (7)	C3—C2—H2A	118.2
O2^i^—K1—O2	150.35 (3)	C2—C3—C4	117.2 (3)
O1^ii^—K1—O2	79.68 (6)	C2—C3—C6	121.1 (3)
O1—K1—O2	44.61 (6)	C4—C3—C6	121.7 (3)
O3^ii^—K1—O2	104.26 (7)	C5—C4—C3	120.0 (3)
O2^iii^—K1—O2	108.66 (3)	C5—C4—H4A	120.0
O3—K1—O2	74.31 (7)	C3—C4—H4A	120.0
C6—O1—K1^v^	109.68 (18)	C4—C5—C1	119.4 (3)
C6—O1—K1	100.52 (17)	C4—C5—H5A	120.3
K1^v^—O1—K1	83.96 (6)	C1—C5—H5A	120.3
C6—O2—K1^vi^	156.4 (2)	O2—C6—O1	125.4 (3)
C6—O2—K1^vii^	113.13 (18)	O2—C6—C3	117.5 (3)
K1^vi^—O2—K1^vii^	82.49 (6)	O1—C6—C3	117.1 (3)
C6—O2—K1	85.69 (17)		
			
O2^i^—K1—O1—C6	134.52 (17)	K1^vi^—O2—C6—K1	−99.4 (5)
O1^ii^—K1—O1—C6	71.04 (18)	K1^vii^—O2—C6—K1	132.06 (13)
O3^ii^—K1—O1—C6	−8.6 (3)	K1^vi^—O2—C6—K1^v^	−163.2 (4)
O2^iii^—K1—O1—C6	−159.19 (19)	K1^vii^—O2—C6—K1^v^	68.29 (14)
O3—K1—O1—C6	−78.86 (18)	K1—O2—C6—K1^v^	−63.77 (7)
O2—K1—O1—C6	−10.37 (16)	K1^vi^—O2—C6—K1^vii^	128.6 (5)
C6^ii^—K1—O1—C6	50.56 (15)	K1—O2—C6—K1^vii^	−132.06 (13)
C6^iii^—K1—O1—C6	−169.28 (16)	K1^v^—O1—C6—O2	−64.9 (3)
K1^ii^—K1—O1—C6	71.04 (18)	K1—O1—C6—O2	22.4 (3)
K1^v^—K1—O1—C6	−108.96 (18)	K1^v^—O1—C6—C3	114.8 (2)
O2^i^—K1—O1—K1^v^	−116.52 (7)	K1—O1—C6—C3	−157.9 (2)
O1^ii^—K1—O1—K1^v^	180.0	K1^v^—O1—C6—K1	−87.29 (11)
O3^ii^—K1—O1—K1^v^	100.40 (15)	K1—O1—C6—K1^v^	87.29 (11)
O2^iii^—K1—O1—K1^v^	−50.23 (6)	K1^v^—O1—C6—K1^vii^	−4.1 (3)
O3—K1—O1—K1^v^	30.09 (7)	K1—O1—C6—K1^vii^	83.2 (2)
O2—K1—O1—K1^v^	98.58 (9)	C2—C3—C6—O2	−4.5 (4)
C6—K1—O1—K1^v^	108.96 (18)	C4—C3—C6—O2	176.1 (3)
C6^ii^—K1—O1—K1^v^	159.52 (7)	C2—C3—C6—O1	175.8 (3)
C6^iii^—K1—O1—K1^v^	−60.32 (6)	C4—C3—C6—O1	−3.7 (4)
K1^ii^—K1—O1—K1^v^	180.0	C2—C3—C6—K1	108.6 (6)
O2^i^—K1—O2—C6	−67.1 (2)	C4—C3—C6—K1	−70.9 (7)
O1^ii^—K1—O2—C6	−81.54 (17)	C2—C3—C6—K1^v^	−125.9 (3)
O1—K1—O2—C6	10.31 (16)	C4—C3—C6—K1^v^	54.7 (4)
O3^ii^—K1—O2—C6	−168.72 (17)	C2—C3—C6—K1^vii^	−47.7 (3)
O2^iii^—K1—O2—C6	41.19 (16)	C4—C3—C6—K1^vii^	132.8 (3)
O3—K1—O2—C6	115.25 (18)	O2^i^—K1—C6—O2	138.37 (12)
C6^ii^—K1—O2—C6	−94.85 (18)	O1^ii^—K1—C6—O2	93.78 (17)
C6^iii^—K1—O2—C6	37.2 (2)	O1—K1—C6—O2	−160.9 (3)
K1^ii^—K1—O2—C6	−116.44 (16)	O3^ii^—K1—C6—O2	13.6 (2)
K1^v^—K1—O2—C6	63.56 (16)	O2^iii^—K1—C6—O2	−141.38 (15)
O2^i^—K1—O2—K1^vi^	89.41 (15)	O3—K1—C6—O2	−60.97 (17)
O1^ii^—K1—O2—K1^vi^	75.00 (7)	C6^ii^—K1—C6—O2	74.81 (17)
O1—K1—O2—K1^vi^	166.84 (12)	C6^iii^—K1—C6—O2	−149.60 (19)
O3^ii^—K1—O2—K1^vi^	−12.18 (9)	K1^ii^—K1—C6—O2	74.81 (17)
O2^iii^—K1—O2—K1^vi^	−162.27 (8)	K1^v^—K1—C6—O2	−105.19 (17)
O3—K1—O2—K1^vi^	−88.21 (8)	O2^i^—K1—C6—O1	−60.7 (2)
C6—K1—O2—K1^vi^	156.5 (2)	O1^ii^—K1—C6—O1	−105.34 (19)
C6^ii^—K1—O2—K1^vi^	61.68 (7)	O3^ii^—K1—C6—O1	174.51 (17)
C6^iii^—K1—O2—K1^vi^	−166.30 (7)	O2^iii^—K1—C6—O1	19.50 (18)
K1^ii^—K1—O2—K1^vi^	40.09 (7)	O3—K1—C6—O1	99.91 (18)
K1^v^—K1—O2—K1^vi^	−139.91 (7)	O2—K1—C6—O1	160.9 (3)
O2^i^—K1—O2—K1^vii^	176.04 (11)	C6^ii^—K1—C6—O1	−124.31 (16)
O1^ii^—K1—O2—K1^vii^	161.63 (11)	C6^iii^—K1—C6—O1	11.28 (17)
O1—K1—O2—K1^vii^	−106.52 (13)	K1^ii^—K1—C6—O1	−124.31 (16)
O3^ii^—K1—O2—K1^vii^	74.45 (11)	K1^v^—K1—C6—O1	55.69 (16)
O2^iii^—K1—O2—K1^vii^	−75.64 (13)	O2^i^—K1—C6—C3	17.7 (6)
O3—K1—O2—K1^vii^	−1.58 (10)	O1^ii^—K1—C6—C3	−26.9 (6)
C6—K1—O2—K1^vii^	−116.8 (2)	O1—K1—C6—C3	78.4 (6)
C6^ii^—K1—O2—K1^vii^	148.31 (12)	O3^ii^—K1—C6—C3	−107.0 (6)
C6^iii^—K1—O2—K1^vii^	−79.67 (12)	O2^iii^—K1—C6—C3	97.9 (6)
K1^ii^—K1—O2—K1^vii^	126.73 (8)	O3—K1—C6—C3	178.4 (6)
K1^v^—K1—O2—K1^vii^	−53.28 (8)	O2—K1—C6—C3	−120.7 (7)
O2^i^—K1—O3—K1^v^	109.38 (9)	C6^ii^—K1—C6—C3	−45.9 (6)
O1^ii^—K1—O3—K1^v^	−105.92 (13)	C6^iii^—K1—C6—C3	89.7 (6)
O1—K1—O3—K1^v^	−29.55 (7)	K1^ii^—K1—C6—C3	−45.9 (6)
O3^ii^—K1—O3—K1^v^	180.0	K1^v^—K1—C6—C3	134.1 (6)
O2^iii^—K1—O3—K1^v^	40.27 (6)	O2^i^—K1—C6—K1^v^	−116.44 (9)
O2—K1—O3—K1^v^	−72.29 (7)	O1^ii^—K1—C6—K1^v^	−161.03 (6)
C6—K1—O3—K1^v^	−51.77 (7)	O1—K1—C6—K1^v^	−55.69 (16)
C6^ii^—K1—O3—K1^v^	−107.38 (9)	O3^ii^—K1—C6—K1^v^	118.82 (8)
C6^iii^—K1—O3—K1^v^	56.64 (7)	O2^iii^—K1—C6—K1^v^	−36.19 (6)
K1^ii^—K1—O3—K1^v^	180.0	O3—K1—C6—K1^v^	44.22 (6)
C2—N1—C1—C5	0.1 (4)	O2—K1—C6—K1^v^	105.19 (17)
C2—N1—C1—C1^iv^	−179.0 (3)	C6^ii^—K1—C6—K1^v^	180.0
C1—N1—C2—C3	−0.4 (5)	C6^iii^—K1—C6—K1^v^	−44.41 (7)
N1—C2—C3—C4	0.7 (5)	K1^ii^—K1—C6—K1^v^	180.0
N1—C2—C3—C6	−178.8 (3)	O2^i^—K1—C6—K1^vii^	173.13 (8)
C2—C3—C4—C5	−0.7 (4)	O1^ii^—K1—C6—K1^vii^	128.54 (9)
C6—C3—C4—C5	178.8 (3)	O1—K1—C6—K1^vii^	−126.1 (2)
C3—C4—C5—C1	0.4 (4)	O3^ii^—K1—C6—K1^vii^	48.39 (12)
N1—C1—C5—C4	−0.1 (5)	O2^iii^—K1—C6—K1^vii^	−106.62 (8)
C1^iv^—C1—C5—C4	179.0 (3)	O3—K1—C6—K1^vii^	−26.21 (8)
K1^vi^—O2—C6—O1	−119.0 (4)	O2—K1—C6—K1^vii^	34.76 (13)
K1^vii^—O2—C6—O1	112.4 (3)	C6^ii^—K1—C6—K1^vii^	109.57 (6)
K1—O2—C6—O1	−19.7 (3)	C6^iii^—K1—C6—K1^vii^	−114.84 (11)
K1^vi^—O2—C6—C3	61.2 (6)	K1^ii^—K1—C6—K1^vii^	109.57 (6)
K1^vii^—O2—C6—C3	−67.3 (3)	K1^v^—K1—C6—K1^vii^	−70.43 (6)
K1—O2—C6—C3	160.6 (2)		

Symmetry codes: (i) −*x*, *y*+1/2, −*z*+1/2; (ii) *x*−1, *y*, *z*; (iii) −*x*+1, *y*+1/2, −*z*+1/2; (iv) −*x*+2, −*y*+1, −*z*+1; (v) *x*+1, *y*, *z*; (vi) −*x*, *y*−1/2, −*z*+1/2; (vii) −*x*+1, *y*−1/2, −*z*+1/2.

### Hydrogen-bond geometry (Å, °)

**Table:**

*D*—H···*A*	*D*—H	H···*A*	*D*···*A*	*D*—H···*A*
O3—H3A···O1^vii^	0.84 (5)	1.93 (5)	2.770 (4)	177 (5)
O3—H3B···O4^iii^	0.84 (6)	2.15 (6)	2.976 (5)	169 (5)

Symmetry codes: (vii) −*x*+1, *y*−1/2, −*z*+1/2; (iii) −*x*+1, *y*+1/2, −*z*+1/2.
